# Buy solar, get cashback: do consumer subsidies described as promotions influence electricity choices?

**DOI:** 10.3389/fpsyg.2023.1155556

**Published:** 2023-10-04

**Authors:** Swen J. Kühne, Ester Reijnen

**Affiliations:** Applied Cognitive Sciences, Psychological Institute, School of Applied Psychology, Zurich, Switzerland

**Keywords:** electricity product choices, consumer subsidies, incentive, solar electricity, gift

## Abstract

**Introduction:**

Most countries want to make the transition to increased or even exclusive use of renewable energy. To achieve this goal, how can individuals be persuaded to use more renewable electricity? For example, does the way energy companies communicate so-called consumer subsidies matter in this regard, and if so, which communication strategy is best? For example, is a monetary promotion (e.g., cashback) better than a non-monetary one (e.g., gift)?

**Methods:**

In a total of four studies (with a total of more than 1700 participants), we investigated what type of promotion most influenced the choice of a renewable energy product, varying, for example, the environmental friendliness of the renewable energy product.

**Results:**

The monetary promotion (e.g., get $35 back through subsidies) appeared to be the most successful; it significantly increased the choice of the renewable electricity product (i.e., between 12–22%). However, this result was only evident when the subsidized renewable product was not the product already preferred by most individuals. Other measures, such as the willingness to pay (WTP), showed no differential effects.

**Discussion:**

Overall, the observed pattern suggests that promoting renewable energy choices, is similar to promoting donations to a charity. Accordingly, the description of the consumer subsidy as a monetary promotion (i.e., cashback or negative labeling) is most effective in terms of promotion. However, the effect of monetary promotions seems to diminish if the subsidized product is already the product preferred by most consumers. Nevertheless, the use of monetary promotions can encourage the transition to renewable energy.

## 1. Introduction

Much has been said about the negative role of fossil fuels in *climate change* and that we must move away from its use to meet *climate targets* (IPCC, 2022). In addition, the war in Ukraine has highlighted the threat posed by global dependence on fossil fuels (Eurostat, 2022). While all of this may have encouraged a movement toward more *local renewable energy* generation (IEA, 2022), the majority of electricity consumed by individuals or households today still comes from coal and other fossil fuels (Ritchie et al., 2022).

How can governments and private institutions help increase the consumption of electricity from renewable energy sources? It is assumed that the more people switch to electricity from renewable energy sources, the higher the demand for electricity from renewable energy sources, which means that more electricity must be generated from renewable energy sources (Markard and Truffer, 2006). However, the question arises as to which interventions can be used to achieve this goal. So far, interventions have focused particularly on shaping the individual *decision-making* environment (see nudging approach by Thaler and Sunstein, 2008). For example, when renewable electricity is preselected “by default,” it is more likely to be selected as a household electricity product (see Kühne et al., 2019). Although *defaults* within and outside the energy sector are considered very effective (Jachimowicz et al., 2019), other changes in the decision-making environment such as the way “consumer subsidies” are presented have hardly been investigated. So far, consumer subsidies have been applied mainly to fossil fuels to keep their price low. The question is, do consumer subsidies also work for renewable electricity and if so, how do they promote its choice?

Consumer subsidies are defined by United Nations Economic Commission for Europe as “… a government action that directly reduces the price of a fuel or energy service to consumers. A consumer subsidy may also take the form of a cross-subsidy, where a below-cost price to one category of consumers is offset by an above-cost price to another.” (UNECE, 2003, p. 11). In that sense, individual choices for renewable energy could be encouraged by having private energy providers change the pricing of their products by offering fossil or nuclear energy at above-cost prices and renewable energy at below-cost prices. For example, the city of Zurich in Switzerland uses consumer subsidies to reduce the price of certified renewable electricity (e.g., solar) by 2 Rp. / kWh (Zürich, 2019). As in this case of the city of Zurich, it is usually not apparent to consumers that the price of the renewable electricity product has been reduced or that they are receiving subsidies^1^.

However, marketing research has shown that if a product discount is described to the consumer in an apparent way, it has a major impact on product sales (see meta-analysis of Santini et al., 2016). Product promotions can be divided into two categories: *monetary promotions* such as rebates (in $ or %), and *non-monetary promotions* such as free gifts, free shipping, or increased package size. Monetary promotions, while proven to be more effective than non-monetary promotions in some studies (Gilbert and Jackaria, 2002; Alvarez Alvarez and Vázquez Casielles, 2005), are regarded with criticism because they affect the reference price (or quality, see Chandran and Morwitz, 2006) in the long run (that is, the price consumers expect or are willing to pay for a particular product, Darke and Chung, 2005). For example, Diamond and Campbell (1989) have shown that in a monetary promotion such as “detergent $12 cheaper - regular price $14” the discount is integrated into the purchase price, lowering the reference price. Specifically, the consumer expects a lower price for detergent in the future (e.g., $11). In contrast, in non-monetary promotions such as “get a free fabric softener sample when buying a detergent for $14,” the free fabric softener is considered an “extra” and hence is not integrated into the purchase price. The assimilation-contrast hypothesis (Sherif and Hovland, 1961) offers one possible explanation for these effects. If the lower sales price of $12 is perceived as a reasonable substitute for the higher regular price of $14, the detergent will be perceived as a bargain (assimilation). In contrast, if the sales price, in this case for the additional fabric softener, is perceived as belonging to a different price category, the sales price is not perceived as a reduction of the regular price and therefore not assimilated (for an overview, see Mussweiler, 2003). Monetary and non-monetary promotions were found to produce different effects not only on sales but also on perceived *product quality* (Darke and Chung, 2005; Chandran and Morwitz, 2006; DelVecchio et al., 2006). While monetary promotions such as discounts led to lower perceptions of product quality, this was not observed for non-monetary promotions such as gifts. It should be noted, however, that in the meta-analysis by Santini et al. (2016) a positive impact on sales was observed, but no differential impact was observed between the two types of promotions.

The question is whether these findings can be transferred directly onto *electricity products*? In particular, can they help to persuade individuals or households to buy an electricity product made from renewable energy sources such as solar electricity? The answer to this question will depend largely on the similarity between the products (e.g., detergents and electricity products). In this respect, electricity products may differ from normal consumer goods in their quality (hereafter value^2^)-price relationship.

In the case of consumer goods, a higher (lower) value (e.g., due to quality) is generally accompanied by a higher (lower) price. Hence, according to standard economics, the consumer should choose or buy a higher-valued product (detergent X) if (a) the value for the perceived product (V_x_) exceeds that of the price (P_x_), and (b) the value-price difference (V_X_- P_x_; the *benefit*) for the higher-value product (X) is larger than that difference (V_y_- P_y_) for the lower-valued product Y (see Formula 1; by Shampanier et al., 2007).

(a) V_X_ > P_X_ and (b) V_X_ – P_X_ > V_Y_ - P_Y_ (1)

A promotion, such as a discount, on the higher-valued product X, increases the likelihood that it will be purchased because the reduced price increases its “*benefit*.” But the benefit can also be increased by, for example, a gift promotion. However, in the case of electricity products, the electricity that comes out of the socket is the same (i.e., has the same quality as a product dimension), regardless of whether it was generated from renewable or fossil energy. Hence, if consumers perceive the two electricity products of equal value, the net benefit for the renewable electricity product (despite the price reduction) is comparatively low compared to the one for fossil energy, and thus should not be chosen more often. Hence, promotions only work to the extent in which differences in value are perceived.

Furthermore, there is another, perhaps even more important, difference between consumer products (albeit environmentally friendly ones) and electricity products. For example, when you buy a consumer product such as an electric car (e.g., Tesla), you signal to those around you that you care about the environment (so-called “green to be seen” phenomenon, see Griskevicius et al., 2010; Brick et al., 2017). On the other hand, buying solar power is not observable by others (and therefore does not give you a green identity), which reduce its attractiveness and thus its purchase. Buying electricity from renewable sources is therefore more an act of conviction, that is, a sense of commitment that motivates (here “intrinsic motivation”) the decision to support the shift toward greener energy production (see Van der Werff et al., 2013).

Therefore, this behavior may be considered more of a form of prosocial behavior (e.g., donating). Studies in the field of prosocial behavior have shown that incentives (extrinsic rewards) rather tend to *decrease* the desired intrinsically motivated behavior. For example, Gneezy and Rustichini (2000, or for similar results see Newman and Shen, 2012), found that schoolchildren who were given an incentive to collect charitable donations collected less money (see Deci et al.’s, 1999, for a meta-analysis on extrinsic rewards on intrinsically motivated behavior). One explanation is provided by the so-called overjustification hypothesis (Lepper et al., 1973), which assumes that once a person considers an intrinsically motivated activity (e.g., donating) as a means to another end (e.g., receiving a gift), that activity is no longer considered an end in itself. This results in the desired activity being shown less frequently^3^ (see also Newman and Shen, 2012, for examples). Another explanation is the *effective hypothesis*, assuming that exhibiting a behavior associated with an incentive may send a negative signal to *third parties*, e.g., “others think I’m only doing this because of the gift” (see Bénabou and Tirole, 2006; Ariely et al., 2009). Ariely et al. (2009) also found that prosocial activities increased when a monetary incentive was provided, but only when the monetary incentive stayed private. Therefore, if the purchase of electricity products is considered an intrinsically motivated behavior, promotions should actually have a negative effect on its choice.

The above discussion of results can be satisfyingly reconciled through the “two worlds” people live in (see Heyman and Ariely, 2004; Ariely, 2008). One is a world where *market norms* prevail, such as when we buy a detergent. Here we expect to immediately receive a product worth the money we paid. The other is a world where *social norms* prevail, such as when we help a friend move house. In such a situation, we do not expect (immediate) reciprocation, nor do we expect to be paid (i.e., there is no financial reward involved). As long as we keep these two worlds separate, everything is fine (e.g., Gneezy and Rustichini, 2000; Heyman and Ariely, 2004), whereas introducing market norms into a social situation causes problems. For example, Gneezy and Rustichini (2000) showed that the introduction of a fine for parents who were late for picking up their children from daycare led to an increase in the number of children who were picked up late. While parents would feel guilty if the caregiver had to stay late if they themselves were late to pick up their child, after the introduction of a fine they would say to themselves, “I can be late because I am paying for the late pickup.”

Investigating how (i.e., no, positive, or negative impact) promotions encourage the choice of renewable energy products, or rather, which promotions (e.g., gifts or rebates) are most effective in this regard, also provides us with valuable insights into which of two worlds (market or social) energy product choices fall into.

We manipulated the type of promotion (gift, cashback, donation, rebate, etc.) and the two electricity products participants could choose from (from products containing nuclear to pure solar products). We measured the choice of the electricity product, as well as the expected additional costs and willingness to pay (WTP) in order to better understand the underlying measures driving choice.

## 2. Study 1

In our first study, we investigated whether and how different types of promotions (e.g., a gift, cashback) influence solar electricity choice. Participants were able to choose between two products: an eco-electricity product and a solar electricity product. The products correspond to the situation in Switzerland, where many electricity providers no longer have fossil electricity in their products for households, as a rapid expansion of solar electricity use is a goal in Switzerland (see Paganini et al., 2022).

### 2.1. Method

#### 2.1.1. Participants

321 participants aged 20 to 54 years old (*M_age_* = 26.0; *SD_age_* = 5.24; 64.5% female) from the ZHAW Zurich University of Applied Sciences (96%) and the greater area of Zurich (4%) took part in this computer-based online study. In terms of income, 55% of the participants earn less than CHF 2′000 per month. As incentive, participants could enter a raffle for an iPad (which a total of 78% did) or, if a student of the ZHAW School of Applied Psychology, receive course credit instead (which 12% overall did). All participants gave informed consent.

#### 2.1.2. Stimulus material, procedure, and design

**Figure 1:**
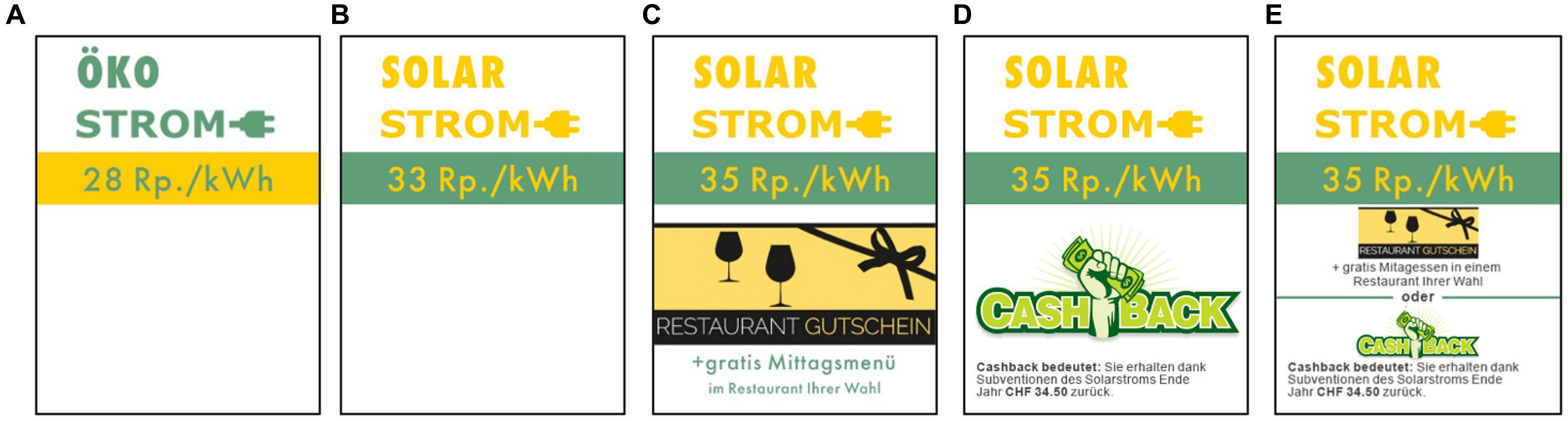
Electricity products used in Study 1, were people had to choose between an eco-electricity product **(A)** and a solar electricity product **(B–D)**. The solar electricity product differed in the conditions: baseline **(B)**, gift **(C)**, cashback **(D)**, and choice between gift and cashback **(E)**. For copyright reasons, the stimuli shown in the illustrations differ slightly from the original Stiumli. Original stimuli are available at https://www.pngall.com/de/cashback-png/download/30420, CC BY-NC.

The *online study* began by asking participants to choose one preference from 3 *leisure time activities* (sports, culture or dine and drink). To disguise the real intent of this question, participants were then asked additional distracting questions (e.g., about sugar-sweetened beverages). Then the main part of the study began, in which participants were told as part of a cover story that they had moved to a new apartment and had to choose between two electricity products (see Figure 1), namely: an eco-electricity product (i.e., a mix of wind, hydro, biogas and solar electricity) with a price of 28 Rp. / kWh and a solar electricity product (i.e., 100% solar electricity). Depending on which condition (baseline, gift, cashback or choice) participants were randomly assigned to, the price of the solar electricity product was different. In the baseline condition, the price shown was net of subsidies (33 Rp. / kWh; see Figure 1B). In all other three intervention conditions, the original price (35 Rp. / kWh) was shown. However, the higher price was compensated by a specific “extra” (worth about CHF 35; rounded value). The gift condition was personalized based on the leisure activity chosen at the beginning (e.g., a free lunch in a restaurant; see Figure 1C)^4^. The cashback condition was a cash amount paid at the end of the year (see Figure 1D). Finally, the choice condition was a personalized gift or cash amount (see Figure 1E), depending on the participant’s choice. After the participants had chosen an electricity product, they were asked a series of questions including: the reason for their choice (open question format), how much more an average Swiss citizen would have to pay to switch from an eco-electricity to a solar electricity product (in CHF per month), how much they would be willing to pay (WTP) for such a switch (in %), their energy behavior (this section included a control question for assessing participants’ attention to the subject), their demographic data (e.g., age, income)^5^, and their intrinsic/extrinsic motivation^6^.

### 2.2. Results

#### 2.2.1. Participants excluded

From the 349 participants who completed the study, 11 participants (3.2%) who answered the control question incorrectly and 3 participants (0.9%) that took part twice were excluded. Furthermore, 14 participants (4.0%), that needed more than 5 min or less than 5 s to complete the electricity product choice task, and those that needed more than 20 min to complete the whole study, were also excluded from the analysis.

#### 2.2.2. Statistics

All statistical analyses were performed using R Statistical Software.

##### 2.2.2.1. Electricity product choice task

**Figure 2:**
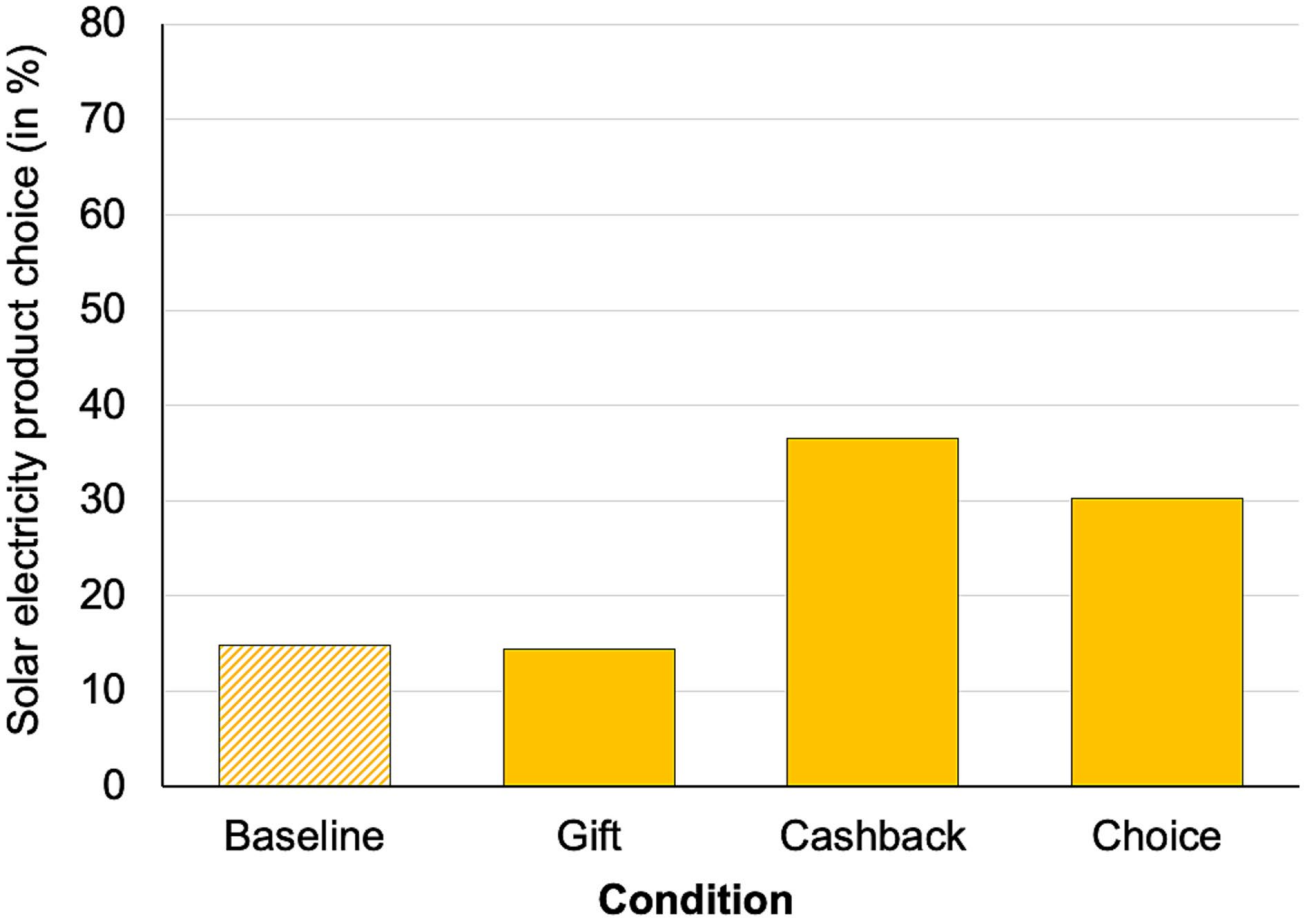
Percentage of solar electricity product choice.

A calculated probit regression model showed a significant main effect of condition, *χ^2^* (3) = 16.43, *p* < 0.001 (see Figure 2), that is, we find a differential effect of “condition” on the probability of choosing the solar electricity product (choice solar in; baseline: 14.8%, gift: 14.5%, cashback: 36.5%, choice: 30.3%). Tukey-adjusted post-hoc tests showed a significant difference between the baseline and the cashback condition (*z* = 3.17, *p* < 0.01; + 21.7%), as well as a marginal significant difference between the baseline and the choice (*z* = 2.38, *p* = 0.08; + 15.5%) condition. Furthermore, there was a significant difference between the gift and the choice condition (*z* = 2.39, *p* = 0.08; + 15.8%), and the gift and the cashback condition (*z* = 3.17, *p* < 0.01; + 22.0%). All other comparisons were insignificant (both *z* < 0.85, *p* > 0.85). Note that in the choice condition, 69.6% of participants decided in favor of the cashback.

##### 2.2.2.2. Expected additional costs (in CHF per month) for switching

A total of 109 participants answered this question (the remaining participants stated that they “do not know”).^7^ Of these 109 participants, those who stated a monetary amount higher than CHF 200.- (*N* = 8) were excluded, as this amount is highly unrealistic (the actual costs are about CHF 10.-).

Although descriptive measures might suggest differences in the stated additional cost between the conditions [baseline: CHF 39.36 (*SD* = 41.64), gift: CHF 47.48 (*SD* = 57.53), cashback: CHF 37.29 (*SD* = 39.55), choice: CHF 46.00 (*SD* = 54.45)], a calculated 1-factorial ANOVA showed no significant main effect of condition, *F* (3, 97) = 0.27, *p* = 0.84.

##### 2.2.2.3. WTP (in %) for switching

From the 321 participants, 4 participants who stated percentages of 200% or higher were excluded.

Again, although descriptive measures might suggest differences in the stated WTP between the conditions [baseline: 10.22% (*SD* = 9.73), gift: 11.82% (*SD* = 14.40), cashback: 14.86% (*SD* = 15.01), choice: 12.48% (*SD* = 12.16)] a calculated 1-factorial ANOVA again showed no significant main effect of condition, *F*(3, 313) = 1.74, *p* = 0.16.

#### 2.2.3. Reasons for choosing an electricity product (eco or solar)

Participants’ reasons for choosing the eco-electricity product were quite diverse, but the most frequently stated reason was that it was the cheaper product (140 statements), followed by the reason that they preferred a *mix of different resources* (71 statements) and the reason that they had concerns about the solar electricity product (37 statements). These concerns included the storage of solar electricity, the amount of sunshine in Switzerland, ecological impact (e.g., disposal of PV panels) and economic aspects. Interestingly, only a few participants mentioned that they did not like the gift (5 statements) or the cashback (1 statements); therefore this was rarely a reason for not choosing solar electricity power.

An equally diverse pattern of reasons was found among participants who chose the solar electricity product. However, almost all of the reasons made some kind of reference to it being the more environmentally friendly option. Again, a few participants stated that they chose the product because they liked the gift (2 statements) or the cashback (10 statements).

### 2.3. Discussion

The *cashback* seems to best promote the choice of solar electricity products. The almost identical effect of the choice can be explained by the fact that 3/4 of participants in this condition chose the cashback. Hence participants seem to prefer *monetary promotions*. We found no significant differences between conditions in terms of expected incremental costs or WTP, suggesting that the promotions at the very least did not have a negative impact on price expectations.

## 3. Study 2

In the second study, we wanted to take a closer look at the following two questions that arose from Study 1: (1) Did the gift option in Study 1 fail because it was not sufficiently personalized? In order to investigate this, we increased personalization of the gift. (2) Is the choice of electricity products a prosocial behavior? To investigate this, we replaced the choice condition with a *donation* condition, which should have no effect on the choice if the behavior is prosocial.

### 3.1. Method

#### 3.1.1. Participants

362 participants aged 19 to 59 years old (*M*_age_ = 26.1; *SD_age_* = 5.92; 59.4% female) from the ZHAW Zurich University of Applied Sciences (86.5%), the University of Basel (10.2%) and the greater area of Zurich (3.3%) took part in this computer-based online study. In terms of income, 58.6% of participants earn less than 2′000 CHF per month. As incentive, participants could enter a raffle for an iPad or a CHF 100 voucher for a grocery store (which a total of 83.7% did) or, if they were a student of the ZHAW School of Applied Psychology, they could choose to receive course credit instead (which 6.9% overall did). All participants gave informed consent.

#### 3.1.2. Stimulus material, procedure, and design

**Figure 3:**
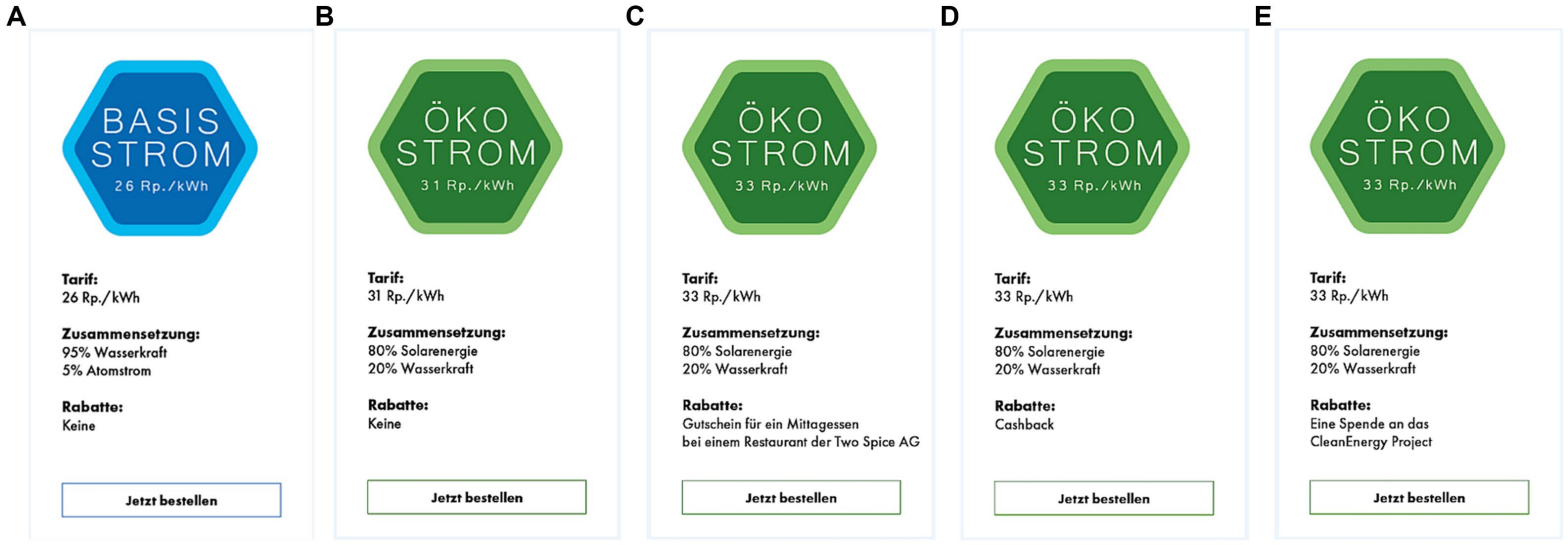
Electricity products used in Study 2, where people had to choose between a basic electricity product **(A)** and an eco-electricity product **(B–D)**. The eco-electricity product differed in the following conditions: baseline **(B)**, gift **(C)**, cashback **(D)**, and donation **(E)**. Situated above the products in the gift, cashback and donation condition was a banner highlighting the promotion of the eco-electricity product.

The stimulus material, procedure and design were similar to Study 1 with the following exceptions: (1) The gift was more personalized. Therefore, to assess participants’ preferred leisure activity, they first had to select one of three overarching categories (sports, leisure activities and shopping). Based on the selected category, they were shown 6 specific activities, such as hiking, swimming, snow sports, cycling, running, and fitness in the sports category. From these they then had to choose their preferred activity, (2) The electricity product options were replaced with basic electricity (i.e., a mix of hydro and nuclear electricity) at a price of 26 Rp. / kWh and eco-electricity (i.e., a mix of hydro and solar electricity) at its original price of 31 Rp. / kWh (i.e., 31 Rp. / kWh in the baseline). The design of the products was also changed (see Figure 3), (3) The choice condition was replaced with the donation condition. By choosing this product, participants were told, they were supporting a solar electricity project in Africa, and (4) Questions were added about how much the gift, cashback or donation is worth to them (in CHF) and how much they approve of it (on a 7-point scale; 1 = strongly disagree, 7 = strongly agree). Finally, the question from Study 1 about what an average Swiss citizen would have to pay to switch from basic to the eco-electricity product (in CHF) was supplemented with the question about how confident they were with their assessment on a 7-point scale (1 = very unsure, 7 = very confident). This question was added because most participants in Study 1 stated that they did not know how much such a switch would cost.

### 3.2. Results

#### 3.2.1. Participants excluded

Out of the 404 participants who completed the study, 6 participants (1.5%) who answered the control question incorrectly and 22 participants (5.4%) who participated twice were excluded. Furthermore, 14 participants (3.5%), who needed more than 5 min or less than 5 s to complete the critical choice task, as well as those who needed more than 20 min to complete the entire study were excluded from the analysis.

#### 3.2.2. Statistics

##### 3.2.2.1. Electricity product choice task

**Figure 4:**
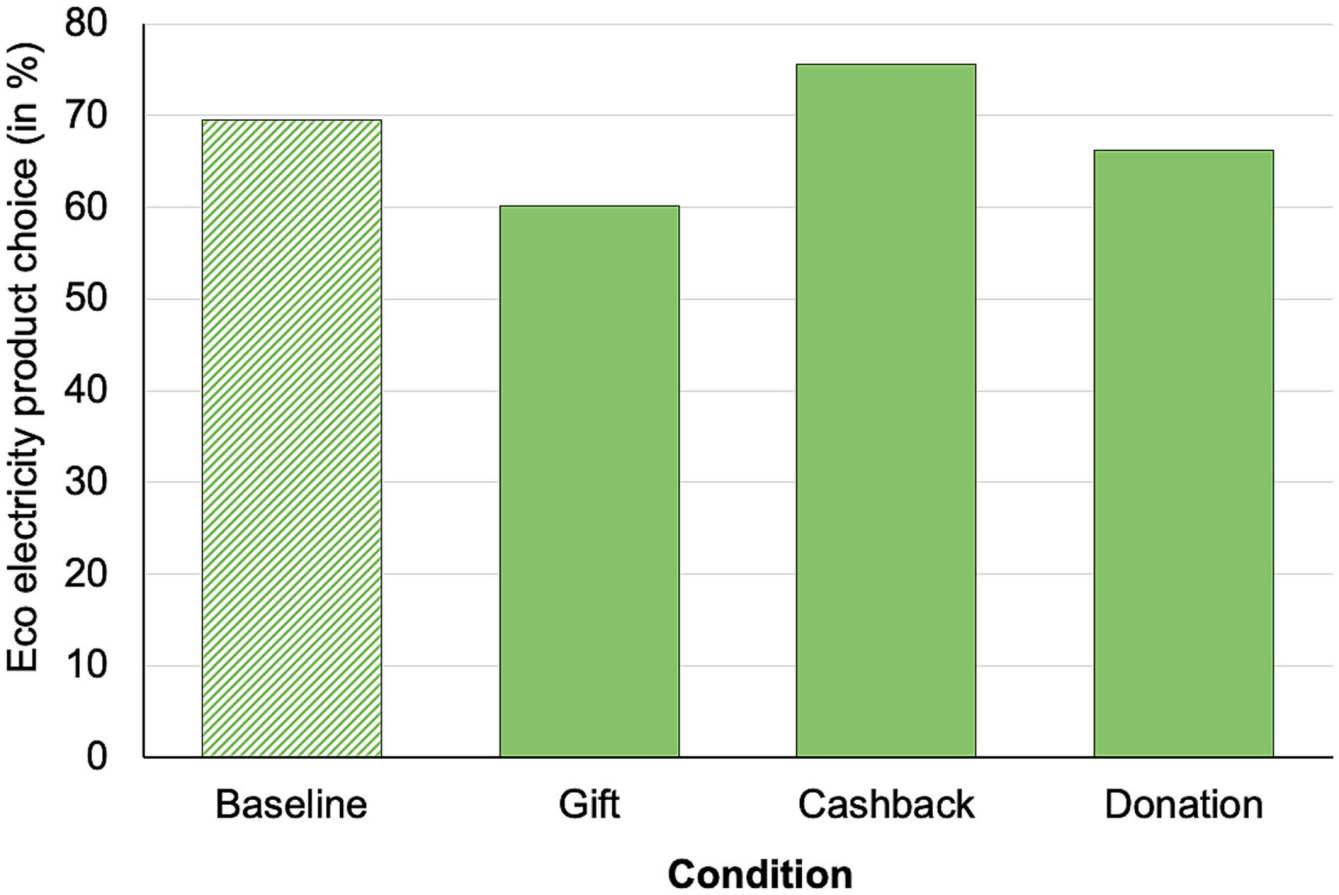
Percentage of eco-electricity product choice.

In contrast to Study 1, a calculated probit regression model showed no significant main effect of condition (choice eco in; baseline: 69.5%, gift: 60.2%, cashback: 75.6%, donation: 66.3%, see Figure 4), *χ^2^* (3) = 5.21, *p* = 0.16.

##### 3.2.2.2. Expected additional costs (in CHF per month) for switching

As in Study 1, participants who stated a monetary amount higher then 200 CHF (*N* = 14; leaving 348 participants) were excluded. Note, 73.9% of participants indicated that they were very to rather unsure about the expected costs.

Although descriptive measures here also might suggest differences in the stated additional costs between the conditions [baseline: 38.42 CHF (*SD* = 48.44), gift: 25.58 CHF (*SD* = 31.17) cashback: 36.45 CHF (*SD* = 41.69), donation: 30.32 CHF (*SD* = 36.57)], a calculated 1-factorial ANOVA again showed no significant main effect of condition, *F* (3, 344) = 1.96, *p* = 0.12.

##### 3.2.2.3. WTP (in %) for switching

The descriptive measures indicate differences in the stated WTP between the conditions [baseline: 17.04% (*SD* = 11.28), gift: 19.06% (*SD* = 18.58), cashback: 24.44% (*SD* = 20.42), donation: 19.13% (*SD* = 16.39)] and here the 1-factorial ANOVA showed a significant main effect, *F*(3, 358) = 3.09, *p* < 0.05. Tukey adjusted post-hoc tests showed a difference between the baseline and the cashback condition, *t*(358) = 2.93, *p* < 0.05, but no other comparison (*t* < 2.15, *p* > 0.14).

##### 3.2.2.4. Value (in CHF) of the promotion and approval

From the 267 participants in the 3 intervention conditions, 3 participants who stated a monetary amount higher than 200 CHF (actual value was 34.50 CHF) were excluded.

As indicated by the descriptive measures for the perceived value of the promotion [gift: 37.00 CHF (*SD* = 33.83), cashback: 28.55 CHF (*SD* = 32.60), donation: 21.17 CHF (*SD* = 41.25)], the 1-factorial ANOVA showed a main effect, *F*(2, 261) = 4.35, *p* < 0.05. The Tukey adjusted post-hoc test indicated a difference in the perceived value of the donation and the gift, *t*(261) = 2.94, *p* < 0.05, but no other comparison (*t* < 1.59, *p* > 0.25).

Similarly, regarding participants’ approval of the promotion [gift: 3.55 (*SD* = 1.85), cashback: 4.47 (*SD* = 1.85), donation: 4.72 (*SD* = 1.58)], the 1-factorial ANOVA showed a main effect, *F*(2, 261) = 11.48, *p* < 0.001. The Tukey adjusted post-hoc tests showed a difference between the gift and the cashback, *t*(261) = 3.59, *p* < 0.001 and between the gift and the donation, *t*(261) = 4.47, *p* < 0.001, but not for the other comparison (*t* < 0.90, *p* > 0.64).

#### 3.2.3. Open question: reason for choosing product

The reasons given for the choice were similar to those in Study 1. For example, the most common reason for choosing basic electricity was that it was cheaper (92 statements). Regarding the choice for eco-electricity: again, the reason given was that it was the more environmentally friendly option (159 statements). There were only a few participants who mentioned the promotions in a negative way, the cashback in particular was mentioned positively.

### 3.3. Discussion

Interestingly, in Study 2 none of the promotions had a different effect on participants’ choice. This could be due to the electricity products that were used. The products in Study 2 are lower in terms of “environmentally friendliness^8^” than those in Study 1 and most people already chose the renewable option as presented in its baseline form.

## 4. Study 3

In Study 1 the *cashback* has an effect. However, is there any way to make the cashback even more effective, by presenting (or framing) it differently? Studies have shown that presenting the rebate not as an absolute number (i.e., CHF saved), but as a relative one (i.e., % saved) is more effective than relative numbers in terms of perceived value of the promotion and purchase intention (see Della Bitta et al., 1981; or González et al., 2016). However, consumers are unaware of their annual electricity consumption or the price they are charged for it (see Clausen, 2008; Tabi et al., 2014; Kühne et al., 2019). This could lead to participants (in Studies 1 and 2) not correctly assessing the appropriateness of the cashback, which compromises the effectiveness of the cashback even though the rebate was presented in an absolute number. What if the cashback is presented not as an amount saved per year, but per unit consumed (here kWh) in an absolute number?

### 4.1. Method

#### 4.1.1. Participants

655 participants aged 18 to 54 years old (*M*_age_ = 24.4; *SD_age_* = 5.07; 61.7% female) from the ZHAW Zurich University of Applied Sciences (91.8%) the greater area of Zurich (8.2%) took part in this smartphone-based online study^9^. In respect to income, 59.1% of participants earn less than 2′000 CHF per month. As incentive, participants could choose to enter a raffle for one iPad (which a total of 90.4% did) or, if they were a student of the ZHAW School of Applied Psychology, they could choose to receive course credit instead (which 4.9% overall did). All participants gave informed consent.

#### 4.1.2. Stimulus material, procedure, and design

**Figure 5:**
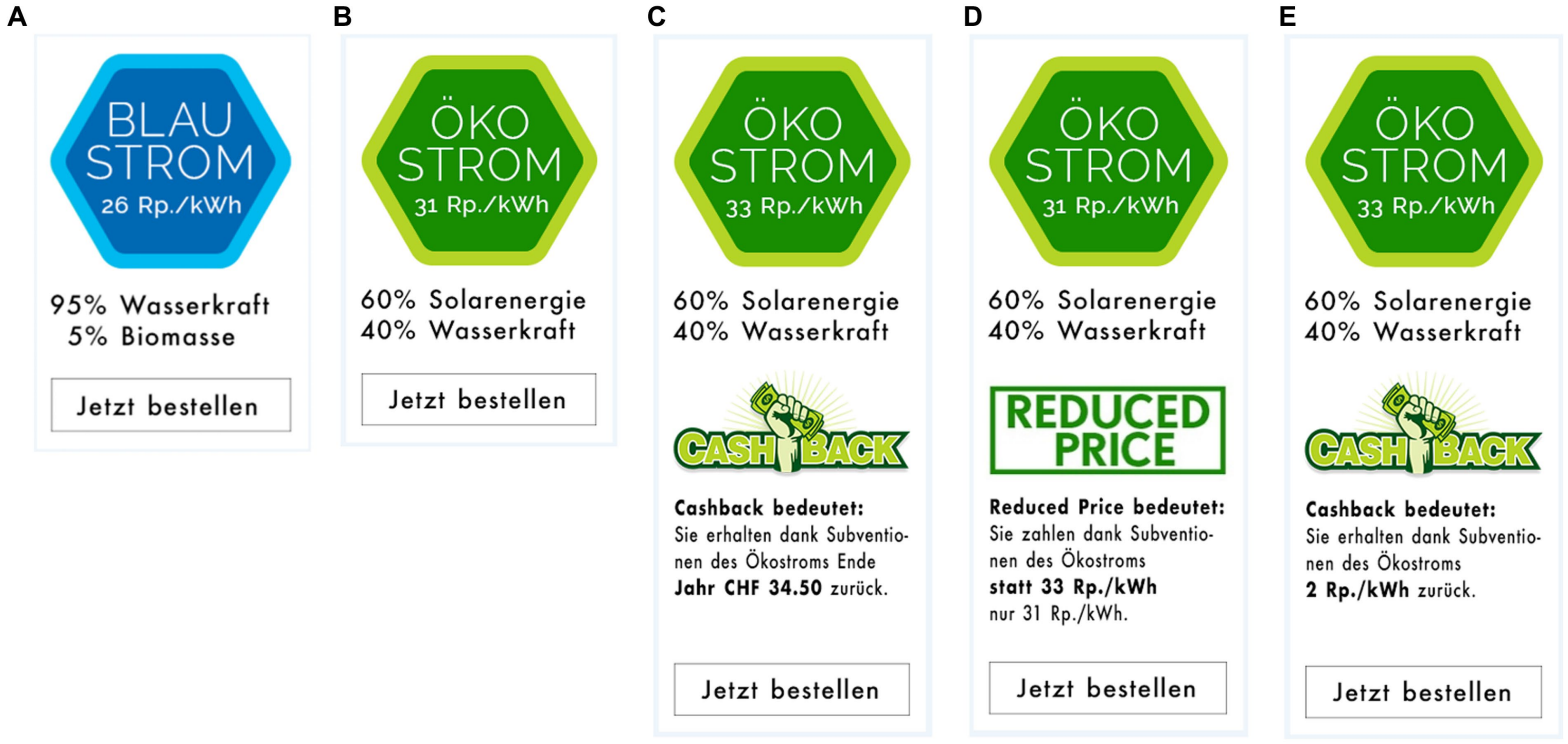
Electricity products used in Study 3, where people had to choose between a blue electricity product **(A)** and an eco-electricity product **(B–D)**. The eco-electricity product differed in the conditions: baseline **(B)**, cashback **(C)**, reduced price **(D)**, and cents back **(E)**. Original stimuli are available at https://www.pngall.com/de/cashback-png/download/30420, CC BY-NC.

The stimulus material, procedure and design were similar to those of Study 1 and 2 with the following exceptions: (1) The electricity product options were replaced with blue electricity (i.e., a mix of 95% hydropower and 5% biomass) at a price of 26 Rp./kWh and green electricity (i.e., a mix of 40% hydropower and 60% solar electricity) at an initial price of 31 Rp./kWh (i.e., 31 Rp./kWh in the baseline; see Figure 5). The former basic electricity product was made slightly more environmentally friendly and changed to the blue power product to avoid “ceiling effects” as observed in Study 2 (however, prices were not changed). (2) While the baseline and cashback conditions remained essentially unchanged (except that they were presented in a different layout), two new conditions were added: *reduced price* and *cents back* promotions. The reduced price condition is another control condition according to Della Bitta et al. (1981), which displays the regular price and the sale price and should fall below the previously used baseline condition in terms of performance. The cents back condition was identical to the cashback condition, but the reduction did not refer to the savings per year (CHF 34.50), but to the savings per unit (2 Rp. / kWh).

### 4.2. Results

#### 4.2.1. Participants excluded

Out of the 733 participants who completed the study, 32 participants (4.4%) who answered the control question incorrectly were excluded. Furthermore, 46 participants (6.3%) who needed more than 5 min or less than 5 s to complete the critical choice task, as well as those who needed more than 20 min to complete the study were excluded from the analysis.

#### 4.2.2. Statistics

##### 4.2.2.1. Binary choice task

**Figure 6:**
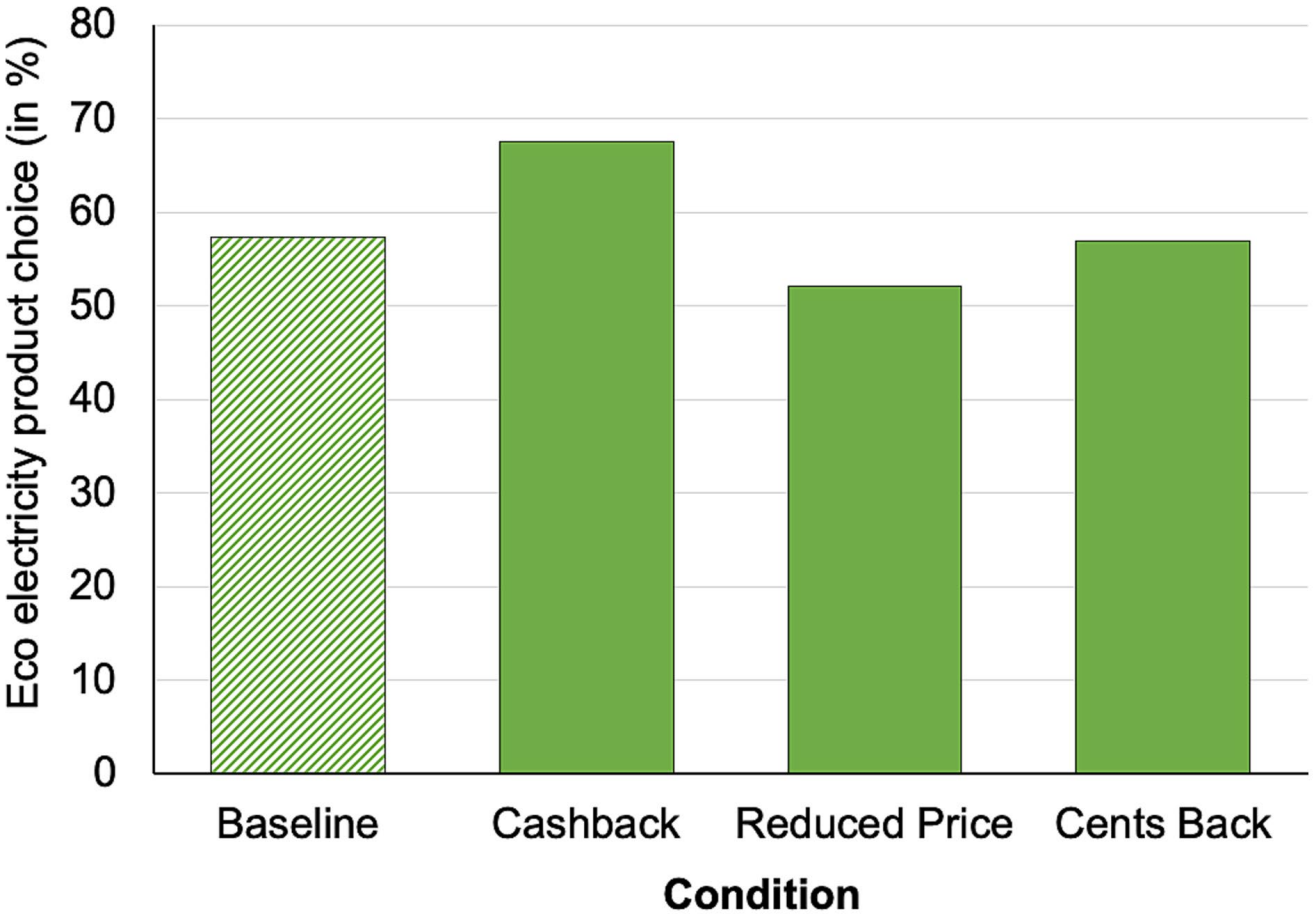
Percentage of eco-electricity product choices.

A calculated probit regression model showed a significant main effect of condition (choice eco in; baseline: 57.3%, cashback: 67.6%, reduced price: 52.1%, cents back: 56.9%, see Figure 6), *χ^2^* (3) = 8.79, *p* < 0.05. Tukey-adjusted post-hoc tests showed a significant difference between the reduced price and the cashback condition (*z* = 2.80, *p* < 0.05, + 15.5%). All other comparisons were insignificant (all *z* < 2.05, *p* > 0.17).

##### 4.2.2.2. Expected additional costs (in CHF per month) for switching

As in the other studies, participants who stated a monetary amount higher than 200 CHF (*N* = 47; leaving 608 participants) were excluded. Note that 81.6% of participants indicated that they were very to rather unsure about the expected costs.

As in the other studies, descriptive measures showed large standard deviations in all conditions [baseline: 47.38 CHF (*SD* = 46.36), cashback: 37.10 CHF (*SD* = 47.33), reduced price: 42.19 CHF (*SD* = 53.52), cents-back: 39.67 CHF (*SD* = 50.03)] and the 1-factorial ANOVA showed no main effect of the condition, *F* (3, 604) = 1.38, *p* = 0.25.

##### 4.2.2.3. WTP (in %) for switching

The descriptive measures indicated differences in the stated WTP between the conditions [baseline: 18.69% (*SD* = 13.78), cashback: 18.05% (*SD* = 12.46), reduced price: 15.70% (*SD* = 16.12), cents back: 15.42% (*SD* = 13.35)] and the calculated 1-factorial ANOVA showed a significant main effect, *F*(3, 650) = 2.74, *p* < 0.05. The Tukey adjusted post-hoc test indicated that there is a marginal significant difference between the baseline and the cents back condition, *t*(650) = 2.43, *p* = 0.07. The other comparisons showed no difference (*t* < 2.24, *p* > 0.11).

##### 4.2.2.4. Value (in CHF) of the rebate and approval

Out of the 463 participants in the 3 intervention conditions, 33 participants who stated a monetary amount higher than 200 CHF (actual value was 34.50 CHF) were excluded.

As indicated by the descriptive measures for the perceived value of the promotion [cashback: 34.80 CHF (*SD* = 21.23), reduced price: 42.29 CHF (*SD* = 51.76), cents back: 29.55 CHF (*SD* = 46.37)] The 1-factorial ANOVA showed a main effect for condition, *F*(2, 427) = 3.22, *p* < 0.05. The Tukey adjusted post-hoc tests showed a difference between the reduced price and the cents back condition, *t*(427) = 2.53, *p* < 0.05, but not for the other comparisons (*t* < 1.60, *p* > 0.25).

Regarding participants’ approval of the promotion [cashback: 4.77 (*SD* = 1.70), reduced price: 4.55 (*SD* = 1.73), cents back: 4.37 (*SD* = 1.84)] the 1-factorial ANOVA showed no main effect for promotion, *F*(2, 427) = 2.06, *p* = 0.13.

#### 4.2.3. Open question: reason for choosing product

This question produced similar results to Study 1 and Study 2, where price (208 statements) was the main reason for choosing the blue electricity product, and sustainability (143 statements) for choosing the eco-electricity product, respectively. All promotions were mentioned as reasons for choosing eco-electricity, but the cashback was mentioned most often.

### 4.3. Discussion

Study 3 replicated the results of Della Bitta et al. (1981) study that absolute price reductions affect choice more than relative reductions. However, as in their study, the differences did not reach significance. Cashback appears to be beneficial only when applied to savings per year. Probably, because it generates a number large enough to make the choice of a higher valued (or more expensive) product worthwhile.

## 5. Study 4

So far, the cashback (compared to the baseline) only had an effect in Study 1, in which participants had to choose between two products, one of which was solar electricity. The question is why? A possible explanation could be provided by the study of Lowe and Barnes (2012), who found a benefit of *monetary promotions* over *non-monetary promotions* not for regular products, but for innovative products (e.g., innovative batteries). In Switzerland, the so-called “regular” electricity product is renewable electricity, which most electricity providers such as the local provider in Zurich offer to households accordingly by default (i.e., blue electricity). Although around 60% of the electricity generated in Switzerland already comes from renewable sources such as hydropower, the share of solar electricity is only about 4% (BFE, 2022). Given Switzerland’s declared goal of phasing out of nuclear energy, this number is too low! (see Paganini et al., 2022). Based on the aforementioned specifics of the Swiss electricity market, we assume that the (pure) solar power product used in Study 1 represents an innovative product. That solar electricity can be considered as an innovative product was also suggested by Hai (2019). To test whether solar electricity is perceived by participants as innovative and thus unfamiliar, we included relevant questions in Study 4.

Another possible explanation could be that, depending on the condition (baseline or cashback), different groups of people were targeted in terms of their motivation for environmentally friendly behavior, leading to differences in the choice of solar power product. For example, it is known that people who are rather *intrinsically* motivated exhibit more environmentally friendly behavior (see De Groot and Steg, 2010; Aitken et al., 2016; Masson and Otto, 2021). On the other hand, Van Dam and van Trijp (2016) claim that so-called *amotivated* people, rather accidentally, if at all, act environmentally friendly, respectively buy environmentally friendly products. Between these two poles of motivation (intrinsic motivation, amotivation) lies extrinsic motivation (see Deci and Ryan, 2000), under which the vast majority of people can be subsumed (see Van Dam and van Trijp, 2016). Since it is assumed that people with a more extrinsic motivation act economically rationally, environmentally friendly behavior – according to van Dam and van Trijp (2016) – can most likely be triggered by external regulation (at the individual or societal level), that is, by rewards or punishments. While our cashback is a regulatory measure that rewards more environmentally friendly choices, negative labeling, for example, can penalize less environmentally friendly choices. An example of negative labeling is the negative eco-label for food, which is only applied to products that are not organic (see Van Dam and De Jonge, 2015). To test whether primarily extrinsically motivated individuals respond to economic incentives (positive / negative), we replicate Study 1 but add a condition with a negative label and simultaneously assess participants’ motivation.

### 5.1. Method

#### 5.1.1. Participants

385 participants aged 18 to 53 years old (*M*_age_ = 25.5; *SD_age_* = 4.97; 68.3% female) from the ZHAW Zurich University of Applied Sciences (98.4%) the greater area of Zurich (1.6%) took part in this smartphone-based online study. In respect to income, 63.9% of participants earn less than 2′000 CHF per month. As incentive, participants could choose to enter a raffle for vouchers (4 × 100 CHF) of a major Swiss grocery store (which a total of 95.8% did) or, if they were a student of the ZHAW School of Applied Psychology, they could choose to receive course credit instead (which 1.3% overall did). All participants gave informed consent.

#### 5.1.2. Stimulus material, procedure and design

**Figure 7:**
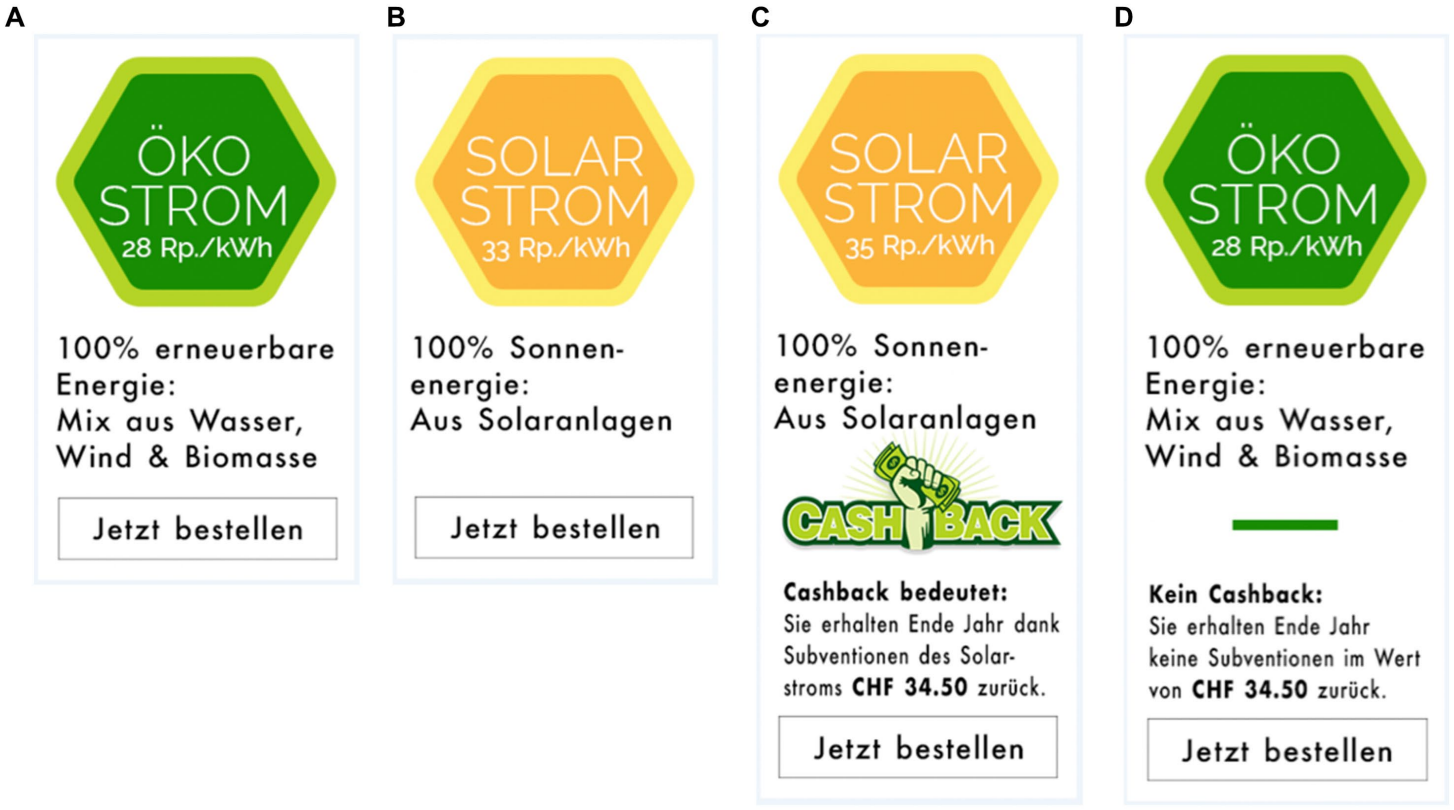
Electricity products used in Study 4, where people had to choose between an eco-electricity product **(A,D)** and a solar electricity product **(B,C)**. The products chosen from differed in the conditions: baseline **(A,B)**, cashback **(A,C)** and negative labeling **(B,D)**. Original stimuli are available at https://www.pngall.com/de/cashback-png/download/30420, CC BY-NC.

The stimulus material, procedure and design were similar to Study 1, with the following exceptions: (1) baseline, cashback and a third condition, called “negative label” was added (the other conditions differed only in their layout from Study 1; see Figure 7), regarding which participants were informed that the eco product was not subsidized, (2) Participants were asked to state their reasons for and against choosing solar electricity. Then, (3) participants had to indicate on a 7-point Likert scale how innovative they found the two products. The question (adapted from: Olshavsky and Spreng, 1996; Moreau et al., 2001; Lowe and Barnes, 2012) were: *How innovative is the electricity product? What influence would the use of the electricity product have on the energy transition? How different is the electricity product from other products you are currently familiar with?* Furthermore, (4) we assessed participant motivation using the MTES (Motivation Toward the Environment Scale; see Pelletier et al., 1998). The MTES items for “external regulation” were supplemented by 4 items. This, because the MTES items only capture the social dimension (e.g., to avoid criticism), but not the economic one as designated by Ryan and Deci (2020). The added 4 items therefore capture financial benefits and punishments as a source of external regulation. In total, participants had to answer 28 items using a sliding scale (0 = strongly disagree, 100 = strongly agree). Last, but not least, (6) participants were asked (at the end of the study) to indicate as precisely as possible the anticipated aim of the study.

### 5.2. Results

#### 5.2.1. Participants excluded

Out of the 416 participants who completed the study, 9 participants (2.2%) who answered the control question incorrectly were excluded. Furthermore, 22 participants (5.3%) who needed more than 5 min or less than 5 s to complete the critical choice task, as well as those who needed more than 20 min to complete the study were excluded from the analysis.

#### 5.2.2. Statistics

##### 5.2.2.1. Binary choice task

**Figure 8:**
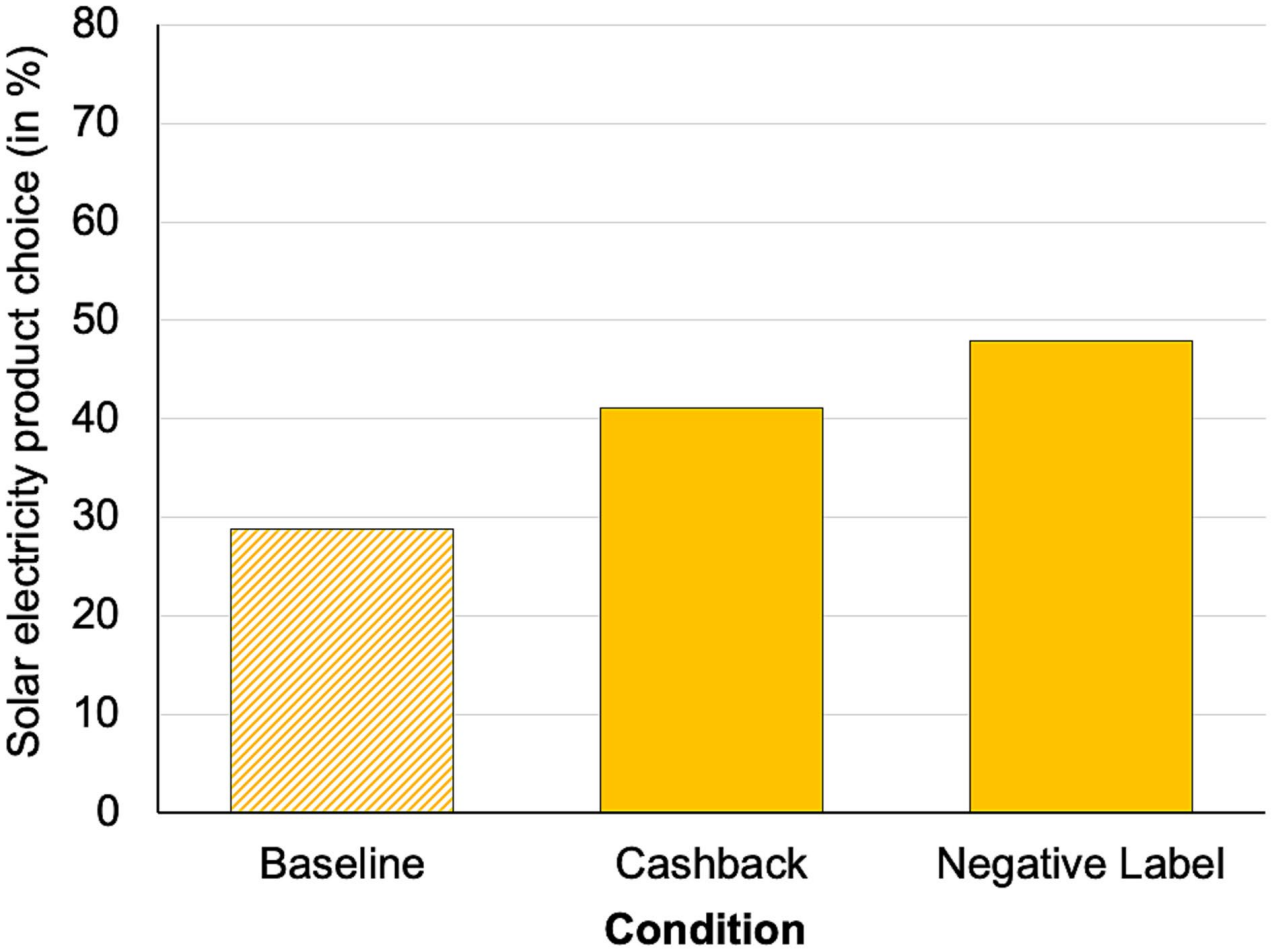
Percentage of solar electricity product choices.

A calculated probit regression model showed a significant main effect of condition (solar electricity choice in; baseline: 28.8%, cashback: 41.1%, negative label: 47.9%, see Figure 8), *χ^2^* (2) = 10.36, *p* < 0.01. Tukey-adjusted post-hoc tests showed a significant difference between the baseline and the negative label condition (*z* = 3.13, *p* < 0.01, + 19.1%) and a marginal significant difference between the baseline and the cashback condition (*z* = 2.11, *p* = 0.09, + 12.3%). However, no significant difference was found between the cashback and the negative label condition (*z* = 1.07, *p* = 0.53).

##### 5.2.2.2. Expected additional costs (in CHF per month) for switching

As in the other studies, participants who stated a monetary amount higher than 200 CHF (*N* = 19; leaving 366 participants) were excluded.

As in the other studies, descriptive measures showed large standard deviations in all conditions [baseline: 47.41 CHF (*SD* = 49.39), cashback: 46.55 CHF (*SD* = 53.07), negative label: 34.05 CHF (*SD* = 41.92)]. A 1-factorial ANOVA showed a marginal main effect of the condition, *F*(2, 363) = 2.78, *p* = 0.06. Thereby the Tukey adjusted post-hoc test indicated a marginal difference between the baseline and negative label condition *t*(650) = 2.13, *p* = 0.06. The other comparisons showed no significant difference (*t* < 1.99, *p* > 0.11).

##### 5.2.2.3. WTP (in %) for switching

The descriptive measures indicate that there are no differences in reported WTP between conditions [baseline: 10.32% (*SD* = 9.29), cashback: 12.83% (*SD* = 12.66), negative label: 12.09% (*SD* = 10.61)], which is confirmed by the non-significant main effect, *F*(2, 382) = 1.88, *p* = 0.15, of a calculated 1-factorial ANOVA.

##### 5.2.2.4. Value (in CHF) of the rebate and approval

Out of the 246 participants in the 2 intervention conditions, 3 participants who stated a monetary amount higher than 200 CHF (actual value was 34.50 CHF) were excluded.

As already evident from the descriptive measures on the perceived value of the two promotions [cashback: 35.32 CHF (*SD* = 16.93), negative label: 35.79 CHF (*SD* = 12.32)], a t-test showed no significant difference between them, *t*(231) = 1.08, *p* = 0.28.

Regarding participants’ approval of the two promotion [cashback: 5.10 (*SD* = 1.42), negative label: 3.61 (*SD* = 1.77)], a t-test showed a significant difference between them, *t*(231) = 7.34, *p* < 0.01. There was more approval of the cashback than the negative label.

Participants were asked if the promotion affected their decision making [cashback: 3.47 (*SD* = 2.00), negative label: 2.99 (*SD* = 1.88)]. The t-test showed a significant difference; *t*(231) = 2.37, *p* < 0.05, indicating, that participants in the cashback condition perceived that their decision was more affected by the promotion than the participants in the negative label condition.

##### 5.2.2.5. Innovativeness of the electricity products

As the descriptive measures indicate (eco: *M* = 4.38, *SD* = 1.09; solar: *M* = 4.37, *SD* = 1.12), there was no difference in perceived innovativeness of the two products, *t*(384) = 0.03, *p* = 0.98.

##### 5.2.2.6. Motivation and electricity product choice

In order to investigate possible (interaction) effects^10^ of motivation with condition on choice, the first step was to see which subscales - according to the results of a hierarchical cluster analysis - could be grouped together^11^. The dendrogram showed a clustering in 2 dimensions. Dimension 1 contains the more internal styles of regulation (hereafter referred to as “internal regulation”): intrinsic regulation, integrated regulation, identified regulation, and introjected regulation. Dimension 2 on the other hand, contains the external and non-regulatory styles (hereafter referred to as “external/non-regulation”): external regulation (original items plus incentive items) and amotivation. The items loaded very well on these two dimensions of motivation (alpha internal regulation = 0.92, alpha external/non-regulation = 0.70).

In a second step, we calculated a probit regression model with choice as the dependent variable and condition, as well as internal and external/non-regulation as predictors. The model showed a significant main effect for condition, *χ^2^*(2, 382) = 10.36, *p* < 0.01, *internal regulation*, *χ^2^*(1, 381) = 4.60, *p* < 0.05, and a marginal effect for *external/non-regulation*, *χ^2^*(1, 380) = 2.89, *p* = 0.09. However, none of the interaction effects were significant (*χ^2^* < 3.85; *p* > 0.14).

To get a clearer picture, we looked at the Pearson correlations for each condition separately. There was a significant correlation of internal regulation with solar electricity choice in the baseline (*r* = 0.19, *p* < 0.05) and marginal significant correlations between external/non-regulation and solar electricity choice in the cashback (*r* = 0.16, *p* = 0.07) and in the negative label condition (*r* = 0.15, *p* = 0.10). The other correlations were non-significant (*r* < 0.11, *p* > 0.22).

#### 5.2.3. Open question: reason for choosing or not choosing solar

Again, price (149 statements) was the main reason for not choosing the solar electricity product, followed by a preference for a mix of electricity sources (66 statements) and concerns about solar electricity (46 statements). The cashback or subsidies were mentioned most often (48 statements) as a reason to choose the solar electricity product, followed by sustainability (30 statements).

#### 5.2.4. Open question: anticipated aim of the study

84% of participants indicated that the goal of the study was to collect some kind of environmental data (e.g., motivations). 11% of the participants stated that they had no idea about the underlying aim of the study or mentioned topics that had nothing to do with it (e.g., concentration). Just 5% “almost” correctly captured the goal of the study (e.g., test the impact of cashback, test nudging techniques, or similar statements).

### 5.3. Discussion

The results of Study 4 are a replication of the results of Study 1, but with the additional finding that the negative label, like the cashback, encourages the choice of environmentally friendly electricity products. In view of our results, the hypothesis that the different results in Studies 1–3 are due to the degree of innovation of the solar electricity product can be rather excluded. The second explanation, that motivation might play a role in the choice, seems to fit better. It appears that – in the baseline condition – participants with high intrinsic motivation were more likely to choose the solar power product (the environmentally friendlier option), consistent with the literature (see De Groot and Steg, 2010; Aitken et al., 2016; Masson and Otto, 2021). On the other hand, intrinsically motivated participants seem to be deterred by the cashback and negative label; at the same time, however, these can attract more participants with external motivation to purchase solar electricity, relatively speaking.

## 6. General discussion and conclusion

Overall, the *cashback* and the *negative label*, both monetary promotions, increased the choice of the more environmentally friendly electricity product by up to 21.7%. A result that is consistent with the finding that *monetary promotions* increase sales more than non-monetary ones in the short term (see Gilbert and Jackaria, 2002; but see the meta-analysis by Santini et al., 2016, for a different result).

Yet, what made these monetary promotions successful? Our first hypothesis was: Its placement on an innovative product (here: solar electricity). Although we cannot completely rule out this hypothesis, the cause is probably different. A closer look at the studies (1 and 4) in which the monetary promotions were effective shows that in these, the proportion of participants who choosing the environmentally friendlier option was very small compared to the other studies (2 and 3). This may suggest that in Studies 1 and 4 we may have created a *conflict* (e.g., of internal goals or values) in the sense that the option preferred by participants was not also the most environmentally friendly. Along these lines, Venema et al. (2020) showed that a nudge – in this case, a social norm (“previously 66% rejected and 34% chose meat”) – led to meat rejection only among participants who signaled that they were ambivalent about meat consumption in general. Other studies also show that nudges are not effective for people with clear preferences (see Theotokis and Manganari, 2015; Trudel et al., 2015; Venema et al., 2019). Returning to our studies: cashback and negative labels can also be viewed as “nudges,” that is, small changes in the decision architecture (see Thaler and Sunstein, 2008) that steer people’s decisions in a particular direction. Therefore, they should be effective primarily in conflict situations, which they are. Our second explanation was that extrinsically motivated people in particular are open to monetary promotions. Indeed, the correlations indicate that participants with high external motivation were more likely to switch to solar electricity in the monetary promotion conditions (cashback and negative label) than in the baseline. In contrast, highly intrinsically motivated participants appear to be deterred by the monetary promotions. That extrinsic rewards undermine intrinsic motivation is consistent with the findings of Deci et al. (1999).

Despite the success of the monetary promotions, are there more effective ones? Now both, cashback and negative label are a type of *rebate* (albeit a small one of 6%). Participants pay more for the renewable electricity per month, but get some of the money back at the end of the year (therefore in our case they can be classified as a nudge). In the domain of donation (e.g., for a charity) rebates are a common measure to increase charity giving: suppose you donate $200 to a charity, and you get a 50% rebate. In this case you would get half of the donated money, $100, returned to you by a third party (for example, the government, through a tax cut at the end of the year). However, rebates have shown to be less effective than so-called matching subsidies (Eckel and Grossman, 2003, 2008; Davis et al., 2005^12^). In the case of *matching subsidies*, if you donate, for example, $100 to a charity, a third party adds a donation of the same amount ($100). Note that with the rebates and the matching subsidies, you end up paying the same amount, $100 (since you got $100 back in the first example). Yet people seem to donate more with matching subsidies (Davis et al., 2005; Eckel and Grossman, 2008). These findings can be explained with the so-called “isolation effect.” In the “donation” problem, information from multiple dimensions, such as the direct consequences for the donor (amount of money donated) or the indirect consequences for the donor or charity (part of the donation is returned), must be integrated. The isolation effect assumes that people tend to disaggregate the dimensions of a multidimensional problem and focus only on the dimension that most directly affects them or that they can control (see Davis, 2006). In the above example, people focus on the amount donated, e.g., $200 under the rebate scheme and $100 under the offset scheme, rather than the total amount donated (e.g., $200). Future studies should therefore investigate whether matching subsidies in the renewable energy sector are more effective than cashback incentives.

At this point, one could critically argue that our results are not generalizable to middle- or high-income individuals, as about half of our participants can be classified as low-income (below CHF 2,000 per month). However, since Blakely et al. (2011) and An (2013), for example, found no relationship between price discounts and household income (or even education) in the food sector, no differential effect is expected with respect to electricity product choice. It could be, however, that home ownership (about 36% in Switzerland, BFS, 2023) is a mediator/moderator between income and electricity product choice.

Last, but not least: Into which world (market norm, social norm) do energy product choices fall? Since the gift had no effect, but the monetary incentive not only influenced extrinsic motivation^13^, but also promoted the choice of environmentally friendly products, we conclude that people do not view energy products differently from common goods (such as detergents) and therefore operate in a *market world* (Ariely, 2008).

Thus, our results appear to be consistent with those found in the donation literature. In this regard, studies showed that small gifts decrease the amount of donation, while monetary incentives increase it (Eckel and Grossman, 2008; Newman and Shen, 2012). Accordingly, donors, are sensitive to changes in their donation price (e.g., due to tax benefits, see Vesterlund, 2006). Therefore, the donation of money, which, however, is to be distinguished from, for example, voluntary work for a charitable purpose, is also to be assigned to the market world.

With respect to the other measures, such as expected switching costs, WTP, and value of promotion, no clear pattern emerged, or consistency with the choice pattern (see Della Bitta et al. (1981), for similar results).

In summary, cashbacks and negative labels are effective in promoting subsidized renewable electricity. However, this is true only if the subsidized product is not the preferred option in the choice situation. Hence, energy providers could use the cashback to accelerate the transition to renewable electricity and thus increase their contribution toward fighting the climate crisis.

## Data availability statement

The raw data supporting the conclusions of this article will be made available by the authors, without undue reservation.

## Ethics statement

Ethical approval was not required for the studies involving humans because it does not fall within the scope of the Human Research Act of the local legislation. The studies were conducted in accordance with the local legislation and institutional requirements. The participants provided their written informed consent to participate in this study.

## Author contributions

SK and ER made an equal substantial, direct and intellectual contribution in all stages of the work. All authors contributed to the article and approved the submitted version.

## Footnotes

^1^Consumers would need to look at the table where the different costs are separated. From the information in this table, it can be deduced that certified renewable electricity results in a refund on the grid taxes, which is a tacit way of subsidizing renewable electricity.

^2^Standing for the overall quality of the product, which we refer to as value in the following to avoid confusion with quality as a product dimension.

^3^Note, we are focusing on promotions or rewards such as gifts which are conditional to showing an intrinsically motivated behavior. Unconditional gifts, which are given to everyone, might foster donation (for an overview see Newman and Shen, 2012).

^4^The gifts were designed so that they would equal the amount of the cashback (CHF 34.50). The amount was calculated using the electricity consumption of a typical 2 person flat in Zurich multiplied by the direct subsidies for solar electricity (refund of grid charge = 2 Rp./kWh).

^5^The demographic data in Study 1 were (as an exception) collected at an earlier time point, that is, after the “distraction” questions.

^6^In Studies 1–3, intrinsic motivation was assessed with three items from van der Werff et al. (2013) and extrinsic motivation with two adapted items from De Young (1986). However, statistical analysis did not reveal a stable pattern across studies, which we attribute to the lack of validity of the items used. We therefore decided not to report the associated results and to use a more elaborate approach to measuring motivation in Study 4.

^7^In this article, switching costs are not about the fees incurred when switching, but about the higher monthly electricity costs incurred when switching to a more environmentally friendly product. In other words, expected switching costs refer to the expected price difference between the less and the more environmentally friendly product.

^8^Note, by a “more or less environmentally friendly” electricity product, we mean that it contributes more or less to the energy transition or to the exclusive use of renewable energy generation in Switzerland.

^9^Studies 3 and 4 were conducted via mobile phones. Since, for example, about 50% of accommodation searches and bookings on Booking.com (2023) are made via mobile phones, this survey method ensures ecological validity. Studies that compared questionnaires using mobile phones and computers as survey methods found no or only minor differences (e.g., longer RT for mobile phones) between these methods (see De Bruijne and Wijnant, 2013; Schlosser and Mays, 2018).

^10^Pearson correlations of the motivational subscales with solar electricity choice are shown in the Supplementary Table S1. We found significant correlations for the subscales: intrinsic regulation (*r* = 0.10, *p* < 0.05), integrated regulation (*r* = 0.13, *p* < 0.01) and external regulation (*r* = 0.14, *p* < 0.01). The correlations of the MTES show a good consistency with the correlations reported by other authors (De Groot and Steg, 2010; Masson and Otto, 2021).

^11^Note, in past studies subscales of the SDT were sometimes collapsed to build brother motivation subscales (e.g., Koestner et al., 2008; Masson and Otto, 2021).

^12^It should be noted that countries differ in terms of the administrative burden of tax deductions for donations, which may reduce the impact of such subsidies (see Peter and Huber, 2021).

^13^We found that intrinsically motivated people are generally more likely to choose renewable electricity products, a result consistent with research by de Groot and Steg (2010).
