# Supplementary material for: Buy solar, get cashback: do consumer subsidies described as promotions influence electricity choices?
Source: Front Psychol. 2023 Oct 4;14:1155556. doi: 10.3389/fpsyg.2023.1155556 (PMC10582937; doi:10.3389/fpsyg.2023.1155556)
Supplement: Supplementary file 1 [file Table_1.DOCX]

Supplementary Material

| Variables | 2. IM | 3. IntR | 4. IdR | 5. IR | 6. ERI | 7. ER | 8. AM |
| --- | --- | --- | --- | --- | --- | --- | --- |
| 1. Solar electricity product choice | .10* | .13** | .03 | .09 | .04 | .14** | -.02 |
| 1. Intrinsic motivation/regulation   (alpha = .87, *M* = 63.0, *SD* = 21.8) |  | .73*** | .66*** | .53*** | .25*** | .10 | -.30*** |
| 1. Integrated regulation   (alpha = .85, *M* = 55.8, *SD* = 23.0) |  |  | .71*** | .62*** | .28*** | .08 | -.33*** |
| 1. Identified regulation   (alpha = .83, *M* = 76.7, *SD* = 16.3) |  |  |  | .61*** | .16 | .03** | -.42*** |
| 1. Introjected regulation   (alpha = .87, *M* = 60.4, *SD* = 22.3) |  |  |  |  | .27*** | .29*** | -.17*** |
| 1. External regulation, plus additional incentive items   (alpha = .66, *M* = 31.4, *SD* = 17.9) |  |  |  |  |  | .24*** | -.06 |
| 1. External regulation   (alpha = .76, *M* = 22.0, *SD* = 16.0) |  |  |  |  |  |  | .27*** |
| 1. Amotivation (non-regulation)   (alpha = .80, *M* = 20.2, *SD* = 16.8) |  |  |  |  |  |  |  |

*** *p* < .05; ** *p* < .01, *** *p* < .001**

**Supplementary Table 1.** Correlation between variables in Study 4 (*N* = 385)
